# BvgR is important for virulence-related phenotypes in *Bordetella bronchiseptica*

**DOI:** 10.1128/spectrum.00794-24

**Published:** 2024-10-04

**Authors:** Maria de la Paz Gutierrez, F. Heath Damron, Federico Sisti, Julieta Fernández

**Affiliations:** 1 Departamento de Ciencias Biológicas, Facultad de Ciencias Exactas, Instituto de Biotecnología y Biología Molecular (IBBM)-CCT-CONICET-La Plata, Universidad Nacional de La Plata, La Plata, Argentina; 2 Department of Microbiology, Immunology, and Cell Biology, Vaccine Development Center at West Virginia University Health Sciences Center, Morgantown, West Virginia, USA; Universita degli Studi Roma Tre, Rome, Italy

**Keywords:** *Bordetella*, type three secretion system, biofilms

## Abstract

*Bordetella bronchiseptica* is a pathogenic bacterium that causes respiratory infections in mammals. Adhesins, toxins, and secretion systems necessary for infection are regulated by the two-component system BvgAS. When the BvgAS system is inactive, there is no transcription of virulence-activated genes, and virulence-repressed genes (*vrg*) are expressed. The regulation of some *vrgs* in *B. bronchiseptica* is dependent upon the virulence-activated gene *bvgR*. Although having a regulatory role, no DNA-binding domain is described for BvgR. Instead, it contains an EAL domain, usually found in cyclic-di-GMP (c-di-GMP)-specific phosphodiesterases. c-di-GMP is a bacterial second messenger that regulates multiple phenotypes in bacteria, including *B. bronchiseptica*. The current study aimed to deepen our knowledge about BvgR. We employed RNA-seq analysis to define the BvgR regulon, and then we investigated the phenotypes in which BvgR regulation might be involved such as biofilm formation, cytotoxicity, and virulence. Our result revealed that BvgR inhibits biofilm formation and flagellin expression in virulent phase. Although BvgR has long been considered a repressor protein, our results show that it also upregulates almost 100 genes. This regulation is likely indirect, as BvgR lacks a DNA-binding domain. Notably, among the upregulated genes, we identified 15 associated with the type three secretion system. Consistent with these findings, a *B. bronchiseptica* strain deficient in *bvgR* was less cytotoxic than the wild-type strain, elicited a milder immune response, and was less able to persist in the lower respiratory tract of mice.

**IMPORTANCE:**

*Bordetella bronchiseptica* is a harmful bacterium responsible for respiratory infections in mammals. Its ability to cause disease is tightly regulated by a system called BvgAS. In this study, we focused on understanding the role of a specific gene called *bvgR* in regulating *B. bronchiseptica*’s virulence factors. Our findings revealed that BvgR, previously thought to primarily repress gene expression, actually plays a complex role in both activating and inhibiting various genes involved in bacterial virulence. This newfound understanding sheds light on the intricate mechanisms underlying *B. bronchiseptica*’s ability to cause infections, providing valuable insights for developing strategies to combat these infections in humans and animals.

## INTRODUCTION

*Bordetella bronchiseptica* is a pathogenic bacterium that causes respiratory infections in mammals. To establish the infection, *B. bronchiseptica* expresses adhesins, toxins, and secretion systems (1). Expression of these virulence factors is regulated by several mechanisms—the *Bordetella* virulence gene (BvgAS) two-component system being the most characterized (1–4). BvgAS is composed of the sensor protein, BvgS, and the transcriptional activator protein, BvgA. BvgS kinase activity is constitutively active under standard culture conditions (37°C). In the laboratory, the addition of modulators, such as MgSO_4_ or nicotinic acid, turns off BvgS kinase activity. However, the signals that regulate BvgS *in vivo* are unknown. Phosphorylated BvgA binds to the promoter of virulence-activated genes (*vags*) and activates their transcription. When the BvgAS system is inactive, there is no transcription of *vags*, and virulence-repressed genes (*vrg*) are expressed (5, 6).

The regulation of *vrg* in *B. bronchiseptica* depends on the product of *bvgR*—a *vag* located downstream of *bvgS* (7). BvgR represses the transcription of some *vrg*, such as flagellin (8). BvgAS regulation has been extensively described, and the function of BvgR remains less understood. To date, microarray analyses have identified some genes controlled by BvgR (9). However, the complete repertoire of proteins modulated by BvgR, as well as the mechanism by which it functions, remains unknown.

Although having a regulatory role, no DNA-binding domain is described for BvgR. Instead, it contains an EAL domain, usually found in cyclic-di-GMP (c-di-GMP)-specific phosphodiesterases (PDE) (7). c-di-GMP is a bacterial second messenger that regulates multiple phenotypes in bacteria, including the transition between motile-to-biofilm lifestyle, virulence, and stress resistance, among other processes (10). Previously, our laboratory showed that high intracellular c-di-GMP levels in *B. bronchiseptica* are associated with biofilm formation and reduced virulence, while low c-di-GMP are linked to a motile planktonic lifestyle (11, 12).

The current study aimed to deepen our knowledge about BvgR. We employed RNA-seq analysis to define the BvgR regulon, and then we investigated the phenotypes in which BvgR regulation might be involved such as biofilm formation, cytotoxicity, and virulence. BvgR has long been considered a repressor protein, but our results show that it also upregulates almost 100 genes. Among these, we found 15 genes associated with the type three secretion system (TTSS). Consistent with the RNA-seq results, a *B. bronchiseptica* strain deficient in *bvgR* was found to be less cytotoxic than the wild-type (WT) strain and exhibited reduced persistence in the lower respiratory tract of mice.

## RESULTS

### BvgR lacks a conserved EAL domain

**Fig 1:**
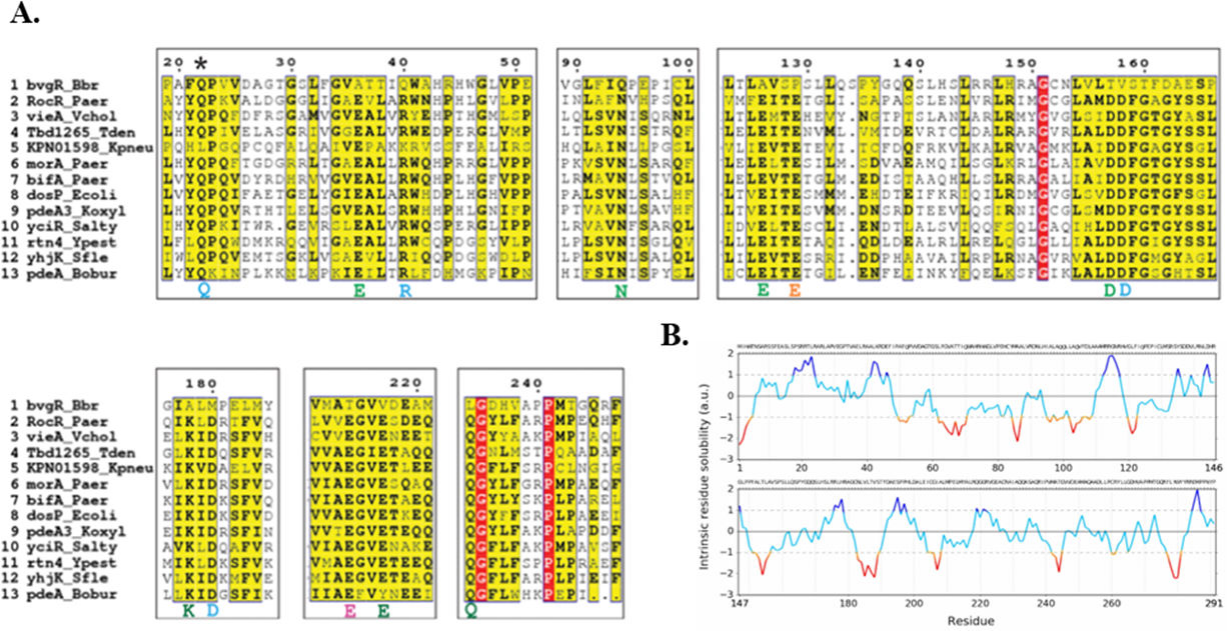
Sequence and surface analysis of BvgR. (**A**) Analysis of the amino acid sequence of BvgR. The amino acid sequence alignment of the EAL domain of BvgR (*B. bronchiseptica*), RocR (*Pseudomonas aeruginosa*), VieA (*Vibrio cholerae*), TBD1265 (*Thiobacillus denitrificans*), KPN01598 (*Klebsiella pneumoniae*), MorA (*P. aeruginosa*), BifA (*P. aeruginosa*), DosP (*Escherichia coli*), PdeA3 (*Klebsiella oxytoca*), YciR (*Salmonella typhimurium*), Rtn4 (*Yersinia pestis*), YhjK (*Shigella flexneri*), and PdeA (*B. bronchiseptica*) were aligned using the software multAlin. (**B**) Prediction of solubility along BvgR extension. Colors ranging from dark blue to red show the intrinsic solubility of each residue. Blue represents the highest values of intrinsic solubility, and red represents the lowest values (Camsol software).

Active PDEs have a consensus sequence that corresponds to the active site and other amino acids important for substrate, ion metal, or water recognition (13). BvgR has been described as an EAL domain-containing protein (7). To begin our study, we aligned the amino acid sequence of the BvgR EAL domain with 12 other EAL domains from active PDEs using the software MultiAling (Fig. 1A). Alignment shows that BvgR lacks the most important residues for PDE activity. There are 13 conserved residues relevant for the PDE activity within the EAL domain (Fig. 1A): four residues that interact with the c-di-GMP molecule (Q, R, D, and D, in light blue), seven residues that interact with the ion metal (E, N, E, D, K, E, and Q, in green), one residue (E, in orange) that stabilizes the loop 6—important for the dimerization of EAL domains—and one residue that coordinates the water molecule for the hydrolytic attack upon c-di-GMP (E, in magenta) (13, 14). BvgR lacks 12 of the 13 conserved residues relevant for activity in the EAL domain. BvgR amino acid sequence has an alanine (A) replacing the glutamate (E) from the EAL motif shown by several authors to be essential for the activity (14–16). It also misses the glutamate that coordinates the catalytic water molecule and presents a degenerated loop 6. Loop 6 of the EAL domain is relevant for PDE activity (17). The only conserved residue that BvgR shares with the other sequences is glutamine (Q) in position 22, which interacts with the substrate (indicated by an asterisk in Fig. 1A). According to the classification of EAL domains proposed by Römling, BvgR belongs to class III: absence of catalytic residues and a degenerated loop 6, so it is predicted to be catalytically inactive (18).

We attempted to purify BvgR in large quantities to test its PDE activity and its ability to bind c-di-GMP *in vitro*. However, purification of this protein was challenging. We used the online tool CamSol to predict protein solubility from the amino acidic sequence, and multiple regions with low solubility were observed (Fig. 1B) (19). Also, *in silico* structure prediction of BvgR revealed several hydrophobic residues located on the protein surface (Fig. S1). For this reason, we cloned the *bvgR* sequence fused to the maltose-binding protein (MBP)—known to enhance solubility—into the plasmid pBBR1MCS5, under the control of a strong promoter (p*nptII*). We expressed BvgR-MBP in a BL21(DE3) CodonPlus strain, engineered to allow high-level expression of heterologous proteins in *Escherichia coli* (20), but were unable to purify the protein in large quantities. Furthermore, no protein was recovered when the MBP tag was cleaved. We sought to express and purify BvgR-MBP from a *B. bronchiseptica* background, hypothesizing that it needs the presence of a *B. bronchiseptica* protein might enhance stability. However, only a small amount of BvgR-MBP was purified from a *B. bronchiseptica* background, preventing *in vitro* assays.

**Fig 2:**
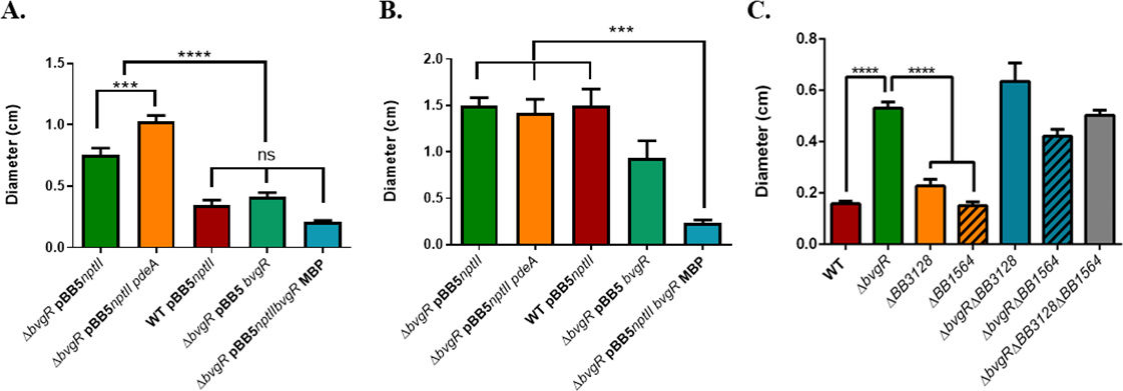
An active phosphodiesterase fails to complement ∆*bvgR* swimming phenotype. (**A**) Swimming motility assays were performed in soft agar plates [Stainer-Scholte medium (SSM) 0.35% (wt/vol) agar; virulent phase conditions] inoculated with a single colony using a sterile toothpick. Swimming diameters were measured after 18 h of incubation at 37°C. (**B**) Swimming motility assay in soft agar plates with the addition of MgSO_4_ (avirulent phase conditions). (**C**) Swimming motility assays performed in soft agar plates of indicated clean deleted mutants, inoculated with a single colony using a sterile toothpick. Swimming diameters were measured after 18 h of incubation at 37°C. Results are shown as mean ± SEM values. Analysis of variance (ANOVA) followed by Tukey’s multiple-comparison tests was used for statistical analysis. The asterisks show statistical significance as follows: ***P ≤ 0.001 and ****P ≤ 0.0001. ns, not significative. Results are based on three biological replicates with at least two technical replicates each. Representative motility agar plates are presented in Fig. S2.

BvgR inhibits *B. bronchiseptica* motility and consequently, a strain lacking BvgR swims in the virulent phase (8). If the inhibition of motility by BvgR is attributed to a presumed PDE activity, introducing a genuine PDE through complementation in *trans* would result in the inhibition of motility in a BvgR-deficient background. To test this hypothesis, we constructed a strain of *B. bronchiseptica* with a clean deletion of *bvgR*, *Bb*∆*bvgR*, that swims in soft agar in the virulent phase. Complementation of *Bb*∆*bvgR* with *bvgR* under its own promoter or with *bvgR-MBP* under the strong promoter *nptII* inhibited the motility in virulent phase (Fig. 2). We hypothesized that overexpression of a known active *B. bronchiseptica* PDE (PdeA) in *Bb*∆*bvgR* would reduce its motility in the virulent phase (12). However, *Bb*∆*bvgR* overexpressing *pdeA* using the pBBR1MCS5*nptII* plasmid showed increased motility compared to *Bb*∆*bvgR*—carrying an empty plasmid—in the virulent phase (Fig. 2A). This result indicates that BvgR cannot be replaced by PdeA. It is important to note that the lack of complementation might be because different PDEs can regulate different phenotypes as some of them only affect local, but not global, c-di-GMP levels as suggested by Hengge (21).

These data suggest that BvgR lacks PDE activity. However, we did not rule out whether BvgR binds c-di-GMP since this molecule is highly flexible, and the protein has one of the key residues for the interaction with the substrate.

When motility was analyzed in avirulent conditions (SS media + MgSO_4_ 40 mM), both the wild-type and mutant strains exhibited motility, without differences between them. When expressing *bvgR* from a plasmid, no inhibition of motility was observed, indicating that other factors, absent in a non-modulated strain, are necessary for BvgR to induce motility inhibition. However, when *bvgR* was expressed as a fusion protein with MBP, motility inhibition was observed (Fig. 2B).

### BvgR modulates both virulence and avirulence factors

**Fig 3:**
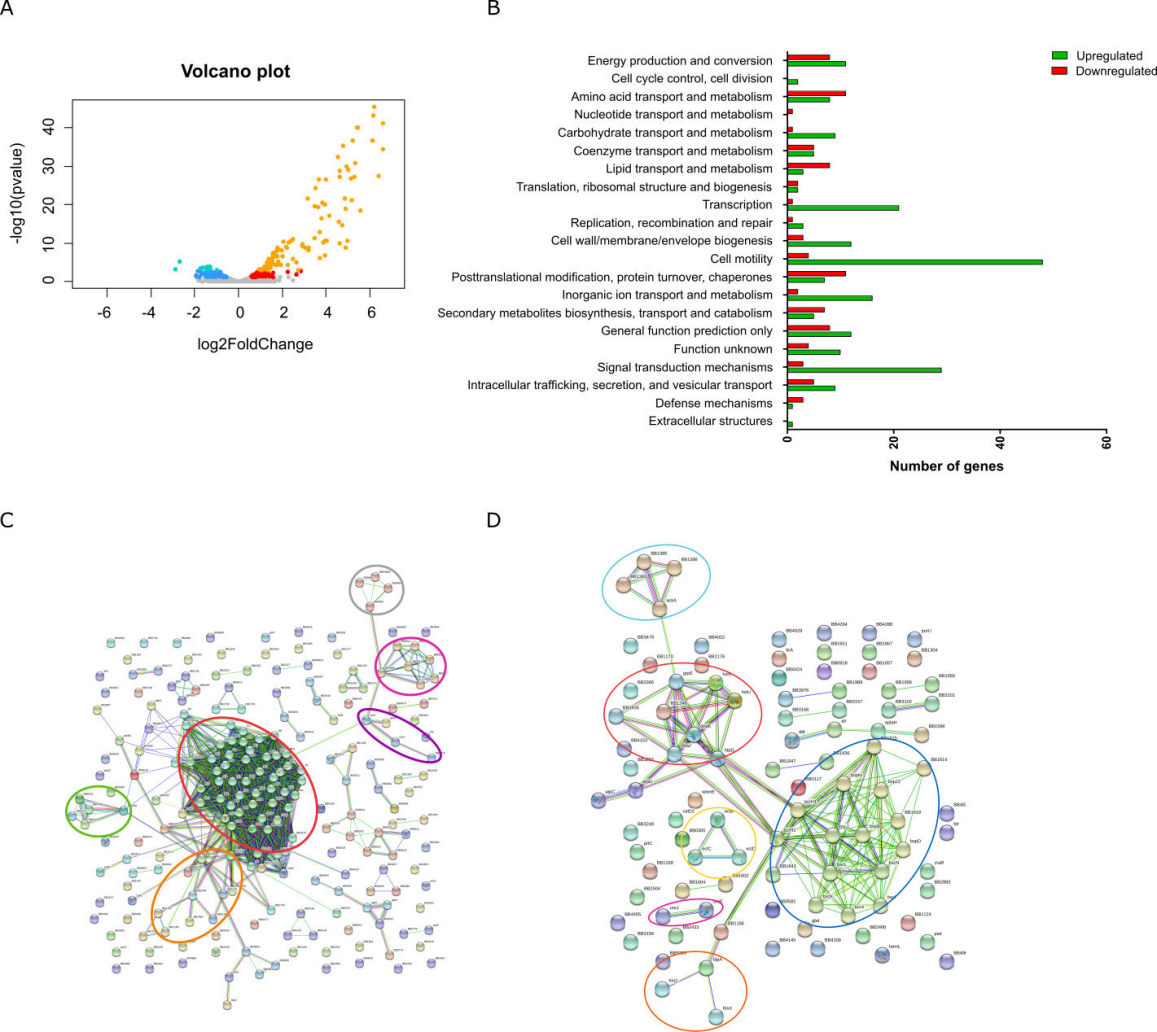
BvgR-repressed and stimulated gene expression in *B. bronchiseptica*. (**A**) Volcano plot of differentially expressed genes between *Bb*WT and *BbbvgR*^−^. Blue and light blue dots represent repressed genes in *BbbvgR*^−^ with *P*-value < 0.05 and corrected *P*-value < 0.05, respectively. Red and orange dots represent activated genes in *BbbvgR*^−^ with *P*-value < 0.05 and corrected *P*-value < 0.05, respectively. (**B**) Clusters of orthologous groups (COGs) analysis of genes regulated by BvgR. (**C**) STRING diagram of genes upregulated in *bvgR* mutant. Circles indicate clusters of genes: chemotaxis and motility (red), c-di-GMP metabolism (orange), transcription (violet), DNA modification (fuchsia), maintenance of lipid asymmetry (green), and non-characterized (gray). (**D**) STRING diagram of genes down-regulated in *bvgR* mutant. Circles indicate clusters of genes: TTSS (blue), chaperones and proteases (red), ectoine biosynthesis (blue), carbohydrate metabolism (light blue), urease (fuchsia), and Fim2, FimX, and BipA adhesins (orange).

Whether BvgR is an active PDE or not, it is involved in motility and probably other phenotypes in *B. bronchiseptica*. To identify the complete regulon of BvgR, we performed RNA-seq from RNA isolated from *Bb* 9.73 wild-type (*Bb*WT) and a *BbbvgR^-^* insertional mutant and compared their transcriptomes. Overall, 319 genes were differentially expressed [*P*-value < 0.05, empirical analysis of digital gene expression (EDGE) test] between those strains, of which 221 were up-regulated and 98 were down-regulated in the *BbbvgR^-^* strain (Fig. 3A; Table S4). The distribution of the differentially expressed genes in the volcano plot shows that *BbbvgR*^−^ presented more genes with higher expression (positive log2Foldchange values) than with lower expression (negative log2Foldchange values) compared to wild type (Fig. 3A). Moreover, genes with the highest fold change in the comparison are over-expressed in *BbbvgR*^−^, most of them related to the motility of *B. bronchiseptica*. For instance, the master regulator of the flagellar operons, *flhD*, has the highest fold change, of 96.8 (Table S4). The high number of up-regulated genes in the mutant is consistent with the proposed role of BvgR as a repressor of *vrg* genes. However, limited data are available on genes up-regulated by BvgR.

To gain further insight into the genes modulated by BvgR, we assigned the differentially expressed genes to Clusters of Orthologous Groups (Fig. 3B). The category with the highest number of genes in the comparison was “cell motility” (22), followed by “signal transduction mechanism” (23), “transcription” (24), “amino acid transport and metabolism” (19), “energy production and conversion” (19), and “posttranslational modification, protein turnover, and chaperones” (18). Around 30 genes were classified as “general function prediction only” or “function unknown.”

According to our reports of the repression of the flagella by BvgR, the *fliC* (*flaA*) gene was up-regulated in the *bvgR* mutant (8). Moreover, the three classes of flagellar operons were highly up-regulated. This evidence suggests that the regulation of BvgR over flagella occurs at the flagellar master operon, *flhDC*. However, FlhDC might not be the only point of regulation by BvgR. Lon, HslU, and HslV were down-regulated in *BbbvgR*^−^ as well. The homologs of these proteases are involved in the regulation of motility by degradation of *fliA* in other bacteria (24, 25).

We performed a protein-protein interaction analysis (STRING) to analyze associations between the differentially expressed genes. The STRING analysis revealed higher expression of two big sets of genes and five small clusters of genes in the *BbbvgR*^−^ strain compared to the WT strain (Fig. 3C). The big clusters, which are connected, are composed of genes from the flagellar and chemotaxis operons (in red) and genes related to the second messenger c-di-GMP metabolism (in orange). Our group already reported that intracellular c-di-GMP concentration regulates *B. bronchiseptica* motility (11). *B. bronchiseptica*’s genome encodes for 22 proteins likely involved in c-di-GMP metabolism, six of which were up-regulated in *BbbvgR^−^*: *bb3128* (that contains an EAL domain), *bb2109* (that contains both EAL and GGDEF domains), *bb2790* and *bb3114* (both contain a GGDEF domain), *bb1564* (that contains HD-GyP), and *bb1561* (containing a PilZ domain). Furthermore, there were some genes located adjacent to or near genes related to c-di-GMP metabolism that were up-regulated as well. That is the case of *bb3115* (located between GGDEF domain-containing protein *bb3114* and EAL domain-containing protein *bb3116*); *bb2662* and *bb2663* (located next to the PDE gene *pdeA-bb2664*); *bb1186*, *bb1190*, and *bb1191* (which belong to the Lap/BrtA system); *bb2108* located upstream of dual EAL-GGDEF domain-containing protein *bb2109* (BB2108 and BB2109 are putative members of a two-component system). *B. bronchiseptica* Lap system proteins were previously reported as *vrg* and repressed by BvgR (26). The other small clusters correspond to proteins associated with transcription (RpoZ, RpsU, and GreA, in purple); to modifications of DNA (BB0906-BB0913, in fuchsia); to the maintenance of lipid asymmetry pathway (BB0163-BB0167, in green) and uncharacterized proteins (BB0483-BB0486 in gray).

The STRING analysis involving the group with lower expression in the *BbbvgR*^−^ compared to the wild-type strain revealed a big cluster composed of TTSS genes (Fig. 3C, in blue). We found several genes within the *bsc* locus of the TTSS with fold changes of around −2 (*bsp22*, *bopBDN*, *bscIJKLN*, *bcrH1*, *bcrH2*, *bb1614*, *bb1615*, and *bb1618*). In addition, we observed five other clusters composed of genes codifying for chaperones and proteases (GrpE, DnaJ, DnaK, HtpG, HslU, HslV, and Lon, in red); for proteins responsible for ectoine biosynthesis (in yellow); for enzymes related to carbohydrate metabolism (in light blue); for urease accessory proteins (in pink); and for FimX, Fim2, and BipA (virulence-related proteins, in orange).

**Fig 4:**
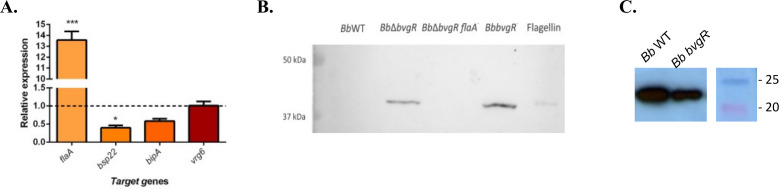
Confirmation of differences observed in the transcriptomic analysis. (**A**) Relative expression of selected genes determined by RT-qPCR. Values were calculated with the 2-^ΔΔ^ method using *recA* as the control expression gene. The experiment was performed at least three times for each gene, and an average of values is presented. Student’s *t*-test was used for statistical analysis. Significant differences are indicated as follows: *P < 0.05; ***P < 0.001. (**B**) Western blot with anti-flagellin polyclonal sera. Whole cells of the indicated strains were normalized by culture OD_650_. (**C**) Western blot with anti-Bsp22 polyclonal sera. Wild-type and mutant-strain supernatants were treated as described in the methods. Precision Plus Ladder (BioRad) was employed as reference to calculate the molecular weight of proteins. Full-size western blots are available in supplemental material (Fig. **S7**).

To confirm the results obtained by RNA-seq, mRNA expression levels of *flaA*, *bsp22*, *bipA*, and *vrg-6* were determined by qRT-PCR in *Bb*WT and *BbbvgR*^−^. In accordance with the RNA-seq analysis, *flaA* expression was 13-fold higher in *BbbvgR*^−^ (Fig. 4A). Both *bsp22* and *vrg-6* mRNA expression correlated with the transcriptomic analysis (Fig. 4A). Although expression of *bipA* was lower in *BbbvgR*^−^ than in *Bb*WT in the RNA-seq results, the mRNA levels measured by qRT-PCR were not different between the strains. In addition, the expression of *flaA* and *bsp22* was determined by western blot in *Bb*WT and *BbbvgR*^−^. The sample composed of *BbbvgR*^−^ lysate showed more flagellin protein than those of *Bb*WT, while the amount of Bsp22 in *BbbvgR*^−^ supernatant was lower (Fig. 4B and C).

### Up-regulation of PDEs is not related to motility inhibition by BvgR

Although it is unlikely that BvgR has PDE activity, it still could be able to modify c-di-GMP intracellular levels through the regulation of proteins involved in the c-di-GMP metabolism, such as diguanylate cyclases (DGCs) or PDEs. As mentioned before, two genes of putative PDEs (*bb3128* and *bb1564*) were expressed more in *BbbvgR*^−^ than in the wild-type strain. *bb3128* encodes for an EAL-containing protein with two transmembrane domains, while *bb1564* encodes for an HD-GYP-containing protein, with one transmembrane domain and one coiled-coil region.

We hypothesized that the overexpression of *bb3128* and *bb1564* in *BbbvgR*^−^ caused a drop in the c-di-GMP levels and consequentially increased motility of the strain, as high PDE activity is frequently associated with motility in different bacteria (27). To test this, we deleted *bb3128* and *bb1564* in a *Bb*∆*bvgR* background and evaluated the motility of the double mutants (*Bb*∆*bvgR*∆*bb3128* and *Bb*∆*bvgR*∆*bb1564*) and the triple mutant *Bb*∆*bvgR*∆*bb3128*∆*bb1564*. We used a clean mutation to avoid possible polar effects when evaluating multifactorial phenotypes such as motility and biofilm. If any of these putative PDEs were involved in the regulation of motility, a strain lacking one or both would not show an increased motility. However, the double mutants and the triple mutant (*Bb*∆*bvgR*∆*bb3128*, *Bb*∆*bvgR*∆*bb1564*, and *Bb*∆*bvgR*∆*bb3128*∆*bb1564*) swam in soft agar like the *Bb*∆*bvgR* strain (Fig. 2C; Fig. S2). On the contrary, the *Bb*WT strain showed total repression of its motility in the virulent phase (Fig. 2C). This result indicates that the repression of the motility performed by BvgR in *B. bronchiseptica* during its virulent phase is not dependent on BB3128 or BB1564.

### BvgR controls biofilm formation in the virulent phase

BvgR represses *B. bronchiseptica* motility, but its regulation of other phenotypes has not been reported (8). Flagellar motility and biofilm formation are commonly co-regulated in other bacteria; therefore, we investigated whether BvgR is required for *B. bronchiseptica* biofilm formation.

**Fig 5:**
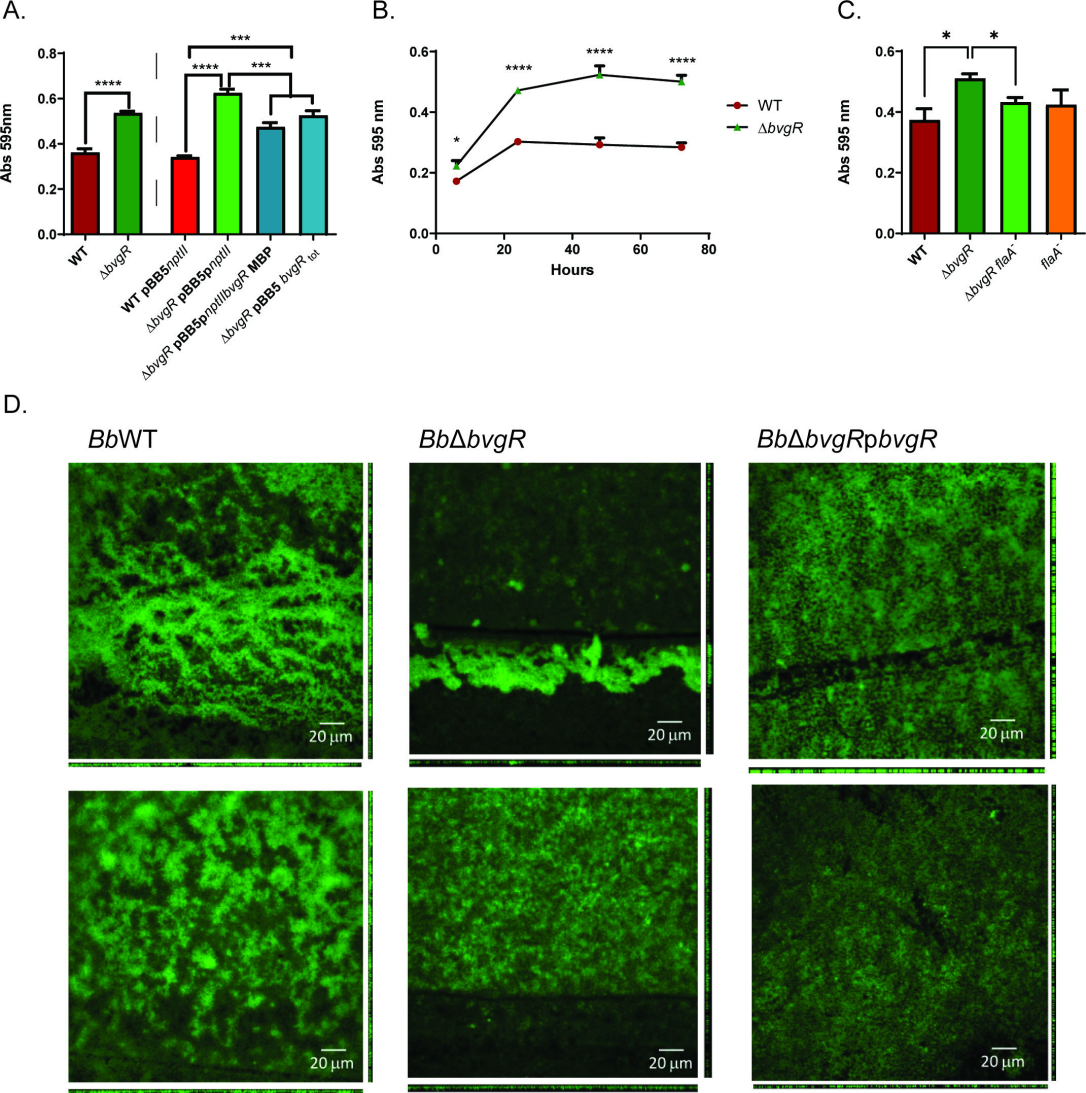
Biofilm formation by wild-type and mutant strains in SSM. In all experiments, wells were washed and stained with 0.1% crystal violet. Remanent crystal violet was resuspended in acetic acid 33% (vol/vol) and quantified by absorbance at 595 nm. (**A**) Results after 24 h of static incubation at 37°C. ANOVA followed by Tukey’s multiple-comparison tests was used for statistical analysis. (**B**) Evolution of biofilm formation of wild-type and *Bb*Δ*bvgR* in a 70 h period. Two-tail Student’s *t*-test was used for comparisons. (**C**) Confocal microscopy of *B. bronchiseptica* GFP strains grown on glass surface. Images were taken after 24 h of static incubation at 37°C. COMSTAT 2.1 analysis in Fig. S3. (**D**) Biofilm formation of *bvgR* and *flaA* mutants after 24 h of static incubation at 37°C. For comparison, ANOVA followed by Tukey’s multiple-comparison tests was used. The asterisks show statistical significance as follows: ***P < 0.001 and ****P < 0.0001. Error bars indicate SEMs. In all cases, results are an average of at least three independent experiments.

We assayed the capability to form biofilm of *Bb*WT and of *Bb*∆*bvgR in vitro* in the virulent phase. We observed an increase in biofilm formation in *Bb*∆*bvgR* compared to the wild-type strain (Fig. 5A). Biofilm formation was restored close to wild-type levels when the mutant was complemented in *trans* either with the *bvgR* under its own promoter or the fusion version *bvgR*-MBP (Fig. 5A).

Although both strains were able to form biofilm, differences between strains were observed as early as 6 h of growth and persisted for 72 h (Fig. 5B). Maximum biofilm formation for both strains was observed at 24 h of incubation. After this time point, we did not observe a significant increase in biofilm mass.

To confirm the differences indicated by the crystal violet (CV) assay, we observed the biofilms formed on a glass surface using confocal fluorescence microscopy. As shown in Fig. 5D, the wild-type strain forms a discontinuous biofilm with areas of higher bacterial density. In contrast, the *bvgR* mutant forms a more uniformly distributed, thicker mass, covering the entire surface (Fig. 5D, lower panel). Image analysis by COMSTAT confirmed higher biomass and average thickness in the *bvgR* mutant (Fig. S3). Furthermore, complementation with *bvgR* expressed from a plasmid restored the wild-type phenotype. Notably, we observed groups of bacteria forming biofilm-like structures in both the wild-type and mutant strains with higher altitudes than average levels. However, these formations were more frequent in the mutant strain, which was validated by the higher maximum thickness found in the mutant, as shown in Fig. 5D, upper panel.

Given that *Bb*∆*bvgR* expresses flagella constitutively and taking into account that *Bordetella* biofilm requires flagella in its early stages (28), we decided to investigate whether the constitutive flagella in the mutant was the reason why *Bb*∆*bvgR* presented enhanced biofilm formation. We performed biofilm assays with both wild-type and *Bb*∆*bvgR* strains with the flagellin gene interrupted with a vector (insertional mutant), *BbflaA*^−^ and *Bb*∆*bvgR flaA*^−^, respectively. Although it was described that flagellin is expressed in the first stages of biofilm formation, its absence was not necessary to form a 24 h biofilm (Fig. 5C). However, the absence of FlaA in the *bvgR* mutant background decreases biofilm formation to wild-type levels (Fig. 5C).

### BvgR activates TTSS and affects cytotoxicity in infected mammalian cells

TTSS is a needle-like structure that allows Gram-negative pathogens, such as *B. bronchiseptica*, to translocate effector proteins into the eukaryotic cell cytoplasm (29). *B. bronchiseptica* induces necrotic cell death in a wide range of mammalian cells *in vitro*, in a TTSS-dependent manner (30).

**Fig 6:**
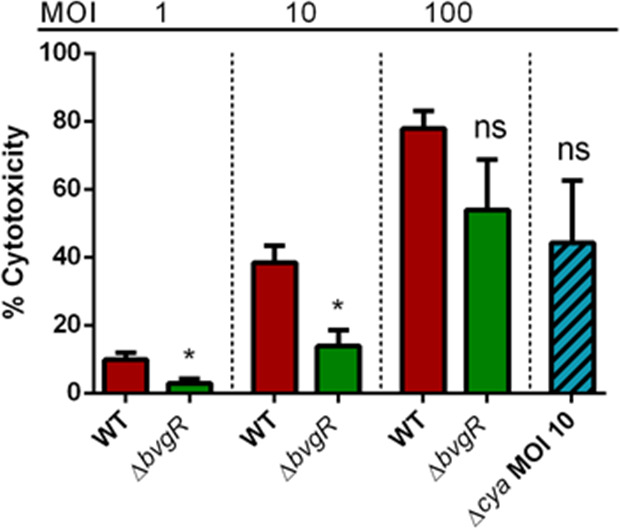
Cytotoxicity of *B. bronchiseptica* in murine macrophages J774.A. J774A.1 macrophages were grown, and bacterial suspensions of *Bb*WT or *Bb*∆*bvgR* were added to the wells in triplicate, at an MOI of 10 and 100. After 4 h of incubation, cytotoxicity was determined by measuring LDH released into a culture medium using Pierce LDH Cytotoxicity Assay Kit. Error bars indicate SEMs. Two-tail Student’s *t*-test was used for comparison. * indicates *P* < 0.05. ns = no significative difference vs *Bb*WT.

Our transcriptomic analysis indicates that BvgR positively regulates TTSS, which to our knowledge has not been observed before. To examine whether BvgR regulation is required for induction of a TTSS-dependent phenotype, J774A.1 mouse macrophage-like cells were infected with *Bb*WT or *Bb*∆*bvgR,* and the ability to induce TTSS-mediated cytotoxicity toward the cells was determined using lactate dehydrogenase (LDH) assay (31). As expected, infection with *Bb*WT resulted in extensive cell death after a 4 h incubation, as the relative amount of LDH released in the extracellular medium was 77% at a multiplicity of infection (MOI) of 100 and 38% at an MOI of 10 (Fig. 6). The cytotoxicity of *Bb*∆*bvgR* infection was significantly reduced as compared with that of *Bb*WT infection, although the attachment showed by both strains was similar (Fig. S4). The cytotoxicity levels induced by *Bb*∆*bvgR* were 20% and 34% of that induced by *Bb*WT at MOI of 100 and 10, respectively. To rule out the possibility that this reduction is due to adenylate cyclase toxin (ACT), a *cyaA*-deficient mutant (RB515) strain was used as a control at an MOI of 10. The phenotype of the *cyaA* mutant was indistinguishable from *Bb*WT (Fig. 6), suggesting that the cytotoxicity measured by this method is associated with the TTSS and not with the AC activity. These results demonstrate that the presence of BvgR is important for the *B. bronchiseptica* TTSS to be fully activated.

### BvgR is required for persistence in the lower respiratory tract of mice

**Fig 7:**
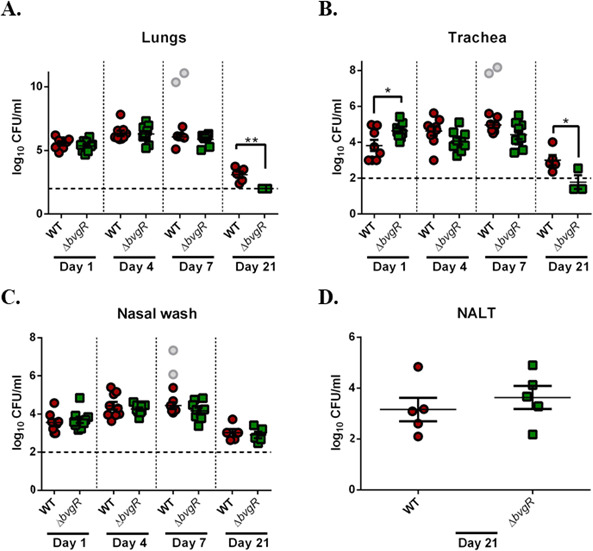
Colonization of murine airway by *Bb*WT and *Bb*∆*bvgR*. CD1 mice were infected with 1 × 10^6^ CFU of *Bb*WT and *Bb*Δ*bvgR*. At 1, 4, 7, and 21 days post-infection, bacterial burdens of lungs (**A**), trachea (**B**), and nasal wash (**C**) were determined. On day 21, bacteria recovered from nasal-associated lymphoid tissue (NALT) were counted (**D**). Each dot represents one animal. Gray dots represent animals found dead. Error bars indicate SEMs. Mann-Whitney *U* test was used for comparisons. The asterisks show statistical significance: *P ≤ 0.05 and **P ≤ 0.01.

Considering that BvgR modulates phenotypes relevant to bacterial pathogenesis, we decided to evaluate the contribution of BvgR regulation on *B. bronchiseptica* virulence in the murine model. Groups of 5-week-old CD1 mice were intranasally inoculated with 1 × 10^6^ CFU of either *Bb*WT or *Bb*∆*bvgR*, and the colonization of the respiratory tract was evaluated on days 1, 4, 7, and 21 after inoculation. Up to 7 days post-infection, similar numbers of CFU were recovered from the lungs, trachea, and nasal cavity of all mice infected with either strain, except for the trachea at day 1, where fewer *Bb*WT bacteria were recovered compared to *Bb*∆*bvgR* (Fig. 7). Two *Bb*WT-infected mice were found dead on day 7 post-infection; tissues were processed for CFU counting but were not considered for statistical analysis (gray dots in Fig. 7). After 21 days of infection, no significant differences were found in bacterial numbers harvested from the nasal cavities of mice infected with either *Bb*WT or *Bb*∆*bvgR* (Fig. 7C and D). In contrast, significantly lower numbers of CFUs were recovered from the trachea of *Bb*∆*bvgR*-infected mice (90% lower) than from *Bb*WT-infected ones 21 days post-infection (Fig. 7B). Specifically, viable *Bb*∆*bvgR* bacteria in the lungs were below the limit of detection of CFU count, even though 2 × 10^3^ CFU of *Bb*WT were recovered from infected lungs (Fig. 7A). These results indicate that *Bb*∆*bvgR* is less efficient than *Bb*WT at persisting in the long term in the lungs and trachea but not in the nasal cavity.

### Effects of BvgR absence in host immune response

The *B. bronchiseptica* TTSS is responsible for part of the immune response during infection. Of note, IL-10, IFN-γ, and IL-17 are regulated by TTSS secreted proteins (23, 32–34). Considering that TTSS is controlled by BvgR, we also evaluated the immune response in mice infected with *Bb*∆*bvgR*.

During the dissection of mice on day 1, we observed that *Bb*WT-infected lungs were swollen, while *Bb*∆*bvgR*-infected lungs looked healthy, similar to control mice. We measured lung weight as an indicator of pulmonary edema, but no differences were found at any timepoint (Fig. S5). There were also no differences in white blood cell counts or lymphocyte counts in the blood between mice infected with different strains throughout the experiment (Fig. S6). We observed differences in neutrophils present in blood quantified by a hematology analyzer, but these differences were not confirmed by flow cytometry (Fig. S6).

**Fig 8:**
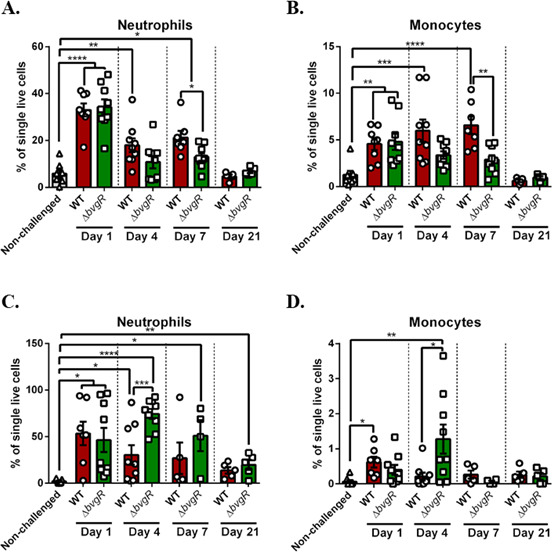
Immune cell quantification during infection. Cells isolated from lung homogenates (**A and B**) and nasal washes (**C and D**) were analyzed by flow cytometry using the LSR Fortessa flow cytometer and analyzed using FlowJo. Results are shown as mean ± SEM values. ANOVA followed by a Tukey’s multiple comparison test was used. The asterisks show statistical significance as follows: *P < 0.05, **P < 0.01, ***P < 0.001, and ****P < 0.0001.

*B. bronchiseptica* induces immune cell infiltration to the lungs during infection. All infected mice presented augmented cell infiltration (Fig. 8). On day 7 post-infection, both neutrophil and monocyte populations were higher in *Bb*WT-infected lungs than in *Bb*∆*bvgR*-infected ones (Fig. 8A and B). Conversely, 4 days post-infection, recruitment of all immune cells analyzed in nasal cavities was higher in mice infected with *Bb*Δ*bvgR* than in those infected by *Bb*WT (Fig. 8C and D).

**Fig 9:**
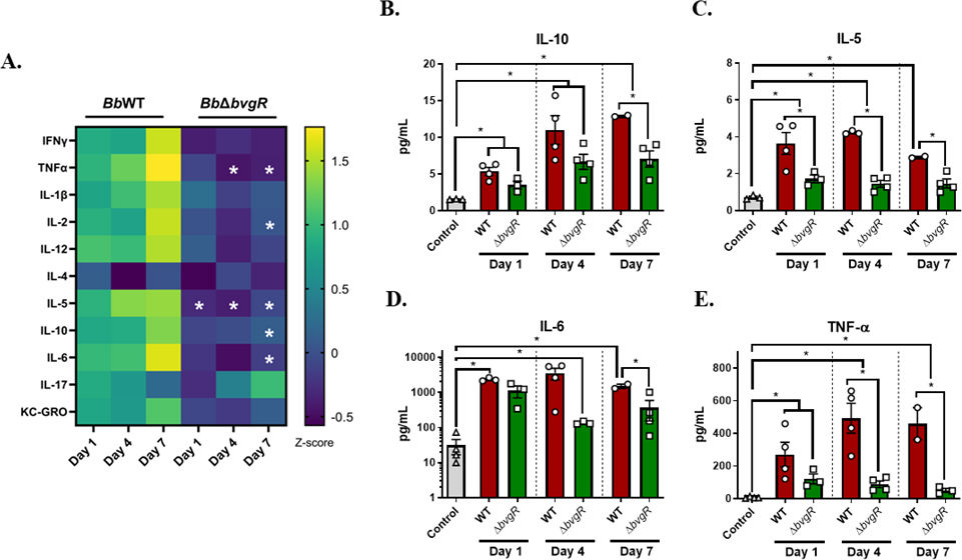
Immune response in *B. bronchiseptica* infected mice. Cytokines in the lung homogenates were determined by a quantitative survey analysis using the Meso Scale Discovery V-PLEX Proinflammatory Panel 1 Mouse Kit. (**A**) Heath map representation of all cytokines analyzed. In detail, analysis is presented for IL-10 (**B**), IL-5 (**C**), IL-6 (**D**), and TNF-α (**E**). Error bars indicate SEMs. For comparisons, ANOVA followed by a Tukey’s multiple comparison tests was used. Asterisk denotes statistical significance, *P < 0.05.

To evaluate the local inflammatory response, cytokine production in the lungs was quantified on days 1, 4, and 7 post-infection (Fig. 9). As expected, *Bb*WT-infected mice presented a strong cytokine response in the lungs compared to control mice, and this was maintained over the course of the experiment. However, mice infected with the *bvgR* mutant showed a different pattern of cytokine secretion compared to the *Bb*WT-infected mice. The anti-inflammatory cytokine IL-10 as well as pro-inflammatory cytokines IL-5, IL-6, and tumor necrosis factor alpha (TNF-α) were significantly lower in mice infected with *Bb*∆*bvgR* (Fig. 9).

## DISCUSSION

In this work, we present a more in-depth study of the BvgR network and phenotypes modulated by this protein. We compared the amino acid sequence of BvgR to catalytically active PDEs and concluded that BvgR belongs to a group of proteins with inactive EAL domains, containing possible c-di-GMP-binding sites. Interestingly, 46 out of the 98 genes down-regulated in the *bvgR* mutant are also down-regulated in a strain of *B. bronchiseptica* overexpressing a DGC (Table S5) (12). This suggests that, despite BvgR not being a phosphodiesterase, it may participate in the c-di-GMP second messenger network.

We also observed that most amino acids predicted to be on the protein surface are hydrophobic, suggesting that BvgR might be associated with other proteins or with the inner membrane of the cell. The presence of a hydrophobic surface may explain the difficulties observed by us and others in purifying BvgR (35). When BvgR was expressed, after being fused to a solubility enhancer like MBP, enhanced motility inhibition was observed. This supports the hypothesis that the localization or availability of BvgR might play a role in BvgR-mediated regulation. Moreover, the overexpression of BvgR-MBP in *B. bronchiseptica* represses motility in the presence of MgSO_4_ (avirulent phase) while the over-expression of *bvgR* (not fused) does not (Fig. 2B). We hypothesize that BvgR shows enhanced solubility by interacting with one or more proteins, which are likely to be expressed in the virulent phase, similar to BvgR. In the absence of those proteins, BvgR cannot exert its function, unless another factor enhances its solubility.

Unable to elucidate the mechanism of action of BvgR by *in vitro* assays, we determined the BvgR regulon by RNA-seq. As expected, the flagellar regulon was derepressed in the mutant. Considering that the master regulator, *flhCD,* was highly up-regulated when BvgR was absent, we propose that BvgR controls motility by inhibiting *flhDC* expression. This is consistent with results presented by Akerley, Cotter, and Miller where they replaced the *flhDC* promotor repressed by BvgR with *fhaB* promotor insensitive to BvgR repression (36). It has been shown that *Bordetella pertussis* can be motile and express flagellum-like structures in a BvgAS-dependent way (37). The flagellar and *flhDC* operon is shared, with minimal differences, between both species. Coutte and colleagues also showed that BvgR regulates this group of genes in *B. pertussis* (9), supporting our results.

Other authors previously suggested that BvgR may act as a PDE, regulating intracellular c-di-GMP levels (9, 38). PDE activity is unlikely for BvgR due to the absence of the catalytic residues needed for PDE activity, and complementation *in trans* of the *bvgR* mutant with a *bonafide* PDE did not restore the wild-type phenotype (Fig. 1A and 2A). Although intracellular low c-di-GMP levels are usually associated with increased motility, we observed that the putative PDEs, whose expression was up-regulated in the *bvgR* mutant, do not regulate motility (27). These results suggest that BvgR inhibits motility by mechanisms independent of BB3128 and BB1564. Since not all PDEs or DGCs are involved in the same phenotype, it is likely that BB3128 and BB1564 regulate other phenotypes (39). Importantly, BvgR lacks known DNA-binding domains, suggesting that its regulation of genes is likely indirect, mediated through other proteins. Moreover, other regulatory systems present in *Bordetella*, such as the response regulator RisA, have been described in *B. pertussis* as important for the regulation of *vags* and *vrgs* (40, 41). Further experiments should consider co-regulation by both two-component systems and c-di-GMP.

Historically, BvgR has been associated with motility in *B. bronchiseptica* and the repression of *vrgs* in *B. pertussis*. It has been reported that vrgs, such as *vrg-6*, *vrg-18, vrg-24*, *vrg-53*, and *vrg-73*, are repressed by BvgR during the virulent phase of *B. pertussis*. However, in our analysis, we observed only the up-regulation of *vrg*-73 in the *bvgR* mutant. Interestingly, 18% and 3% of the differentially expressed genes identified in this study are absent in *B. pertussis* and *Bordetella parapertussis* genomes, respectively. This leads us to hypothesize that some sets of genes modulated by BvgR may be species-specific.

Previous transcriptomic reports using microarray technology showed only a few genes positively regulated by BvgR in *B. pertussis*, which is unsurprising given the negative regulator function assigned to this protein (9). Using a more sensitive technique like RNA-seq, we found an important number of genes that are significantly down-regulated in *B. bronchiseptica bvgR* mutant. Notably, we observed the down-regulation of genes that encode for the TTSS, as well as three adhesins (BipA, Fim2, and FimX). To our knowledge, this is the first description of BvgR as a positive regulator of a virulence factor. In this work, we observed that BvgR is also involved in phenotypes directly related to pathogenesis, such as biofilm formation and cytotoxicity. The absence of BvgR stimulates biofilm formation in the virulent phase in a flagella-dependent manner (Fig. 5C). Nicholson and colleagues previously described the involvement of flagella in biofilm formation (28). Visualization of biofilm under the microscope showed more empty spaces in the wild type than in *Bb*Δ*bvgR*. The mutant, which expresses flagella, fails to form biofilm in the Z direction; instead, the bacteria occupy all available space, resulting in a fully covered surface. Further studies should focus on understanding the specific contributions of flagella to this process. We also observed that non-physiological flagella expression in the virulent phase is responsible for a higher biofilm formation. Although constitutive expression of flagella is not expected in the wild-type strain in virulent phase, we speculate that transient expression of the flagellar system, controlled by BvgR, may be important to promote biofilm formation.

Interestingly, biofilm formation in the Δ*bvgR* mutant is enhanced despite the down-regulation of *bipA* and *fim2*. BipA has been described as an important adhesin for biofilm formation in *Bordetella holmesii* (42). Regarding fimbriae, Irie and colleagues demonstrated that deletion of the fimbriae locus *fimBCD* is detrimental to biofilm formation in *B. bronchiseptica* (43).

*B. bronchiseptica* displays cytotoxicity against mammalian cells in a TTSS-dependent manner (30, 44). The TTSS is a complex machinery, composed of a large number of proteins, and is regulated by many factors in *Bordetella* (29). TTSS genes have been classified according to their regulation and expression by Ahuja (45). We observed that all genes belonging to the *bsc* locus were down-regulated in the *bvgR* mutant. These data suggest that BvgR is needed to efficiently express the needle apparatus. Moreover, in the absence of BvgR, the TTSS activity is impaired as the *bvgR* mutant showed lower activity *in vitro* compared to the wild-type strain (31, 46, 47).

Over the last few decades, other authors have studied the ability of a *bvgR* mutant strain to colonize the respiratory tract of mice (8, 23, 48). In line with our results, they observed no differences in bacterial burden in the lungs between mice infected with the mutant and the wild-type strain during the initial days of infection. Previously, Akerley et al. observed that a *B. bronchiseptica* strain ectopically expressing flagella was cleared from the lungs of rats 15 days post-infection (36). Moreover, they did not detect antibodies against flagella in rats infected with wild-type bacteria, suggesting that *B. bronchiseptica* does not express flagella *in vivo*, and BvgR should be active during infection to repress *flhDC* expression. Differences in the infection process between a Δ*bvgR* strain expressing flagella and the strain that ectopically expresses flagella could be attributed to different levels or temporal activation of the flagella apparatus proteins. The *Bb*Δ*bvgR* strain exhibited motility in the virulent phase, although not as pronounced as that of the modulated wild-type strain, indicating that regulators other than BvgR are involved and may play a role during infection.

Interestingly, we observed that the *bvgR* mutant did slightly better at colonizing the trachea on the first day of infection. We hypothesize that more *Bb*Δ*bvgR* bacteria attach to the tracheal epithelium due to the higher capacity of biofilm formation of the strain. This difference was not persistent during the infection, and the mutant was cleared from the lower respiratory tract (lungs and trachea) 21 days post-infection. The inability to persist in the trachea after 20 days post-infection was also observed in mice infected with TTSS mutants (31). Nicholson and colleagues observed the same difficulty to maintain an infection by a TTSS mutant in pigs after 28 days post-infection (46). Interestingly, the clearance of *Bb*Δ*bvgR* from the lower respiratory tract was observed despite the fact that a lower number of immune cells were recruited to the lungs. There could be different reasons for this to happen, but it is likely that the mutant is more susceptible to the immune response than the wild type, and therefore, it has difficulty persisting in the lungs. Regarding the nasal cavities, we observed that the absence of BvgR was not detrimental during the infection. Persistence of *Bb*Δ*bvgR* strain was observed, even though nasal cavities received a significantly higher number of immune cells 4 days post-infection compared to wild-type-infected mice (Fig. 7 and 8). It is possible that the enhanced biofilm formation by *Bb*Δ*bvgR* helps establish and maintain the infection in the upper respiratory tract. We speculate that while immune cells in nasal cavities were not able to eliminate the bacteria, neutrophils and monocytes were able to prevent dissemination to the lungs, where the mutant strain was less effective at persisting.

The lower recruitment of immune cells in the lungs was consistent with an overall milder immune response induced by *BbΔbvgR*. To our knowledge, this is the first time the cytokine profile associated with a *bvgR* mutant is described. Notably, TNF-α and IL-10 were significantly lower in the lungs of mice infected with *BbΔbvgR*. Early elicited TNF-α is critical for the innate immune response against *B. bronchiseptica*, while IL-10 inhibits bacterial clearance from mice lungs (49, 50). Other authors reported that IL-10 response is dependent on TTSS activity (33, 51). *B. bronchiseptica* strains lacking BscN, a central ATPase necessary for TTSS function, induce lower IL-10 levels in macrophages *ex vivo* or lungs *in vivo*. Pilione and Harvill showed that TTSS inhibits IFN-γ production by splenocytes during infection (50). Although splenocytes were not analyzed in the present work, we saw no differences in IFN-γ production in the lungs. It is noteworthy to mention that IL-8, the main cytokine secreted in response to *B. bronchiseptica* flagellin, was not quantified in the lung supernatants (22). We hypothesize that while a major part of the immune response toward *BbΔbvgR* may be explained due to the absence of TTSS, other BvgR-regulated factors may also play a role.

Always described as a repressor of avirulent factors, BvgR has been relegated from the pathogenesis picture. However, the present work describes BvgR’s prominent role in *Bordetella* pathogenesis. The implication of BvgR up-regulation of TTSS is highlighted from other BvgR-controlled phenotypes. Further studies are needed to unveil how BvgR is connected to other systems to regulate the TTSS in *Bordetella*.

## MATERIALS AND METHODS

### Bacterial strains and growth conditions

Bacterial strains and plasmids are listed in Tables S1 and S2 (supplemental material is found at http://sedici.unlp.edu.ar/handle/10915/168456). *B. bronchiseptica* strains were grown at 37°C either on Bordet-Gengou agar (BGA, Difco) supplemented with 10% (vol/vol) defibrinated sheep blood or in modified Stainer-Scholte medium (SSM), supplemented with gentamycin 50 µg/mL or kanamycin 80 µg/mL, when appropiate. An insertional mutant (*BbbvgR*^−^) was employed in RNA-seq experiments. To avoid possible excision of the inserted plasmid, the strain was always grown in antibiotic presence. In experiments where the antibiotic selection was not possible like in animal or cell experiments, a clean deletion mutant was employed (*Bb*Δ*bvgR*). All experiments were performed using *B. bronchiseptica* grown in SSM without the addition of MgSO_4_, unless explicitly indicated otherwise. When used, 40 mM of MgSO_4_ was added to the media as a phase modulator.

For RNA isolation, bacteria growing on BGA plates for 48 h at 37°C were transferred to SSM supplemented with gentamycin 50 µg/mL and incubated for 14 h at 37°C, shaking at 160 rpm.

*E. coli* (DH5α and S17-1) strains were cultured with lysogeny broth (LB) in a test tube or on solidified LB with 1.5% agar. When appropriate, antibiotics were added to the medium at the following concentrations: 10 µg mL-1 gentamicin and 50 µg mL-1 kanamycin.

Replicative plasmids were introduced to *E. coli* by electroporation using standard techniques. Non- and replicating plasmids were introduced into *B. bronchiseptica* by conjugation. The yeast strain InvSc1 (*Saccharomyces cerevisiae*; Invitrogen) was routinely cultured on YPD medium. When selecting for plasmids carrying the URA3 gene, yeast was grown on YNB with a complete supplemental mixture minus uracil.

### Strain and plasmid construction

All oligonucleotide primers used in the study are listed in Table S3. Cloning was performed by *in vivo* recombination in yeast, as described by Shanks (52). Briefly, plasmid based on the pMQ30 allelic replacement vector was used to generate knockout strains. Two stretches of homologous DNA flanking the genomic region to be deleted were amplified by PCR, named F1 and F2, utilizing primers with 30 or more extra bases to facilitate recombination with adjacent fragments. Yeast cloning techniques were employed to introduce the two fragments in the pMQ30 plasmid to yield pMQ30F1F2. Plasmid recovered from yeast was electroporated in *E. coli* S17-1. All constructions were verified by PCR and DNA sequencing. Allelic replacement mutants were made as previously described by us (53). Briefly, after introducing the plasmid into wild-type *B. bronchiseptica* by biparental conjugation, we selected simple recombinants with antibiotics (streptomycin and gentamicin). Clones resistant to both antibiotics were grown in SSM without antibiotics and plated on LB with sucrose [11% (wt/vol)] to select clones that had lost the plasmid. Clones were double-checked for gentamicin sensitivity and confirmed for deletion by PCR using primers flanking the deleted region.

When needed, plasmids were introduced into *B. bronchiseptica* by conjugation with *E. coli* S17-1, and transconjugants were selected on BGA 80 µg mL^−1^ gentamicin and 200 µg mL^−1^ streptomycin. For a detailed protocol of *B. bronchiseptica* conjugation, see reference (53).

### Motility assays

*Bordetella* motility was determined by measuring the diameter of the migration zone in SSM soft-agar [0.35% (wt/vol) agar] motility plates. Briefly, single colonies grown in BGA were used to inoculate SSM soft-agar motility plates. After 18 h of incubation at 37°C, the diameter of the migration zone was measured. When indicated, the motility plates were supplemented with 40 mM MgSO_4_. Experiments were repeated at least three times, with three technical replicates each.

### RNA isolation and sequencing

*B. bronchiseptica* grown in SSM as described above was pelleted and resuspended in RNA protect bacterial reagent (Qiagen), pelleted again, and stored at –80°C. Three biological replicates were prepared for each strain.

RNA preparation from bacteria was performed as described previously. Briefly, RNA was extracted from the cell pellets using the RNA snap method and purified using RNeasy Mini Kit (Qiagen), as specified by the manufacturer’s instructions. The samples were treated with TURBO DNA-free Kit (Ambion), according to the manufacturer’s instructions for rigorous DNase treatments, and RNA was re-isolated using RNeasy columns. The resulting RNA was quantified on a Qubit 3.0 fluorometer and was checked to be DNA-free by qPCR.

### RNA-seq and analysis

Total RNA was treated with a “Ribo zero kit” (Illumina) for rRNA depletion, and the libraries were prepared using ScriptSeq (Illumina). The libraries were sequenced on an Illumina HiSeq at the Marshall University Genomics Core facility.

RNA-seq analysis was performed using CLC Genomics Workbench 9.5.4. A total of 25 million 2 × 150 bp reads were devoted to each sample. All reads were trimmed based on quality scores with standard settings. All reads were mapped to the *B. bronchiseptica* RB50 reference genome (RefSeq no. NC_002927.3). Only paired reads were counted toward expression analysis. An EDGE was performed to determine differentially expressed genes (54). Genes displayed were filtered such that they must be significant (EDGE test, *P*-value < 0.05) compared to *Bb*WT.

### qRT-PCR

cDNA was synthesized using 500 ng of RNA as a template, gene-specific reverse primers for each target, and M-MLV reverse transcriptase (Promega), according to the manufacturer’s instructions. qPCR mixtures were set up with Excella SYBR Green PCR master mix (Worldwide Medical Products), per manufacturer’s instructions using 1 µL of cDNA. Three technical replicate reactions were run per target gene per sample on a Step One Plus qPCR thermocycler (Applied Biosystems). All primers used are listed in Table S1. Primers were designed on Primer3 and checked for specificity by PCR (55). Gene expression was normalized with *recA*, which is constitutively expressed, using the 2^−ΔΔCt^ method. For statistical analysis, the ΔCt of the three biological replicates with three technical replicates each was calculated, and a Student’s *t*-test was performed.

### Biofilm formation assays

*B. bronchiseptica* biofilm assay was performed as previously reported by our group (56). Cultures with OD_650_ of 0.1 were prepared by resuspending colonies grown in SSM 1.5% agar supplemented with 15% (vol/vol) defibrinated sheep blood into SSM. One hundred microliter of those cultures was pipetted into a 96-well U bottom microtiter plate (polyvinylchloride) and incubated statically at 37°C for 24 h. Planktonic bacteria were removed by washing, and the attached bacteria were stained with 0.1% CV solution. The stain was solubilized in 120 µL of 33% acetic acid solution. Biofilm formation was quantified by measuring OD at 595 nm of 100 µL of the solubilized stain solution. When used, nicotinic acid was added at the indicated concentrations. Experiments were repeated at least three times, with four technical replicates each.

### Confocal microscopy

Cultures of bacteria expressing GFP with OD_650_ of 0.1 were prepared by resuspending colonies grown in SSM 1.5% agar supplemented with 15% (vol/vol) defibrinated sheep blood into SSM. Five milliliter of those cultures was pipetted into a 50 mL conical tube with a cover slip and incubated statically at 37°C for 24 h. Planktonic bacteria were removed by washing, and the attached bacteria were fixed with glutaraldehyde solution [2.5% (vol/vol)]. For the detection of GFP fluorescence, confocal microscopy was performed using an inverted SP5 confocal microscope (Leica Microsystems). GFP was excited using 488 nm laser, and emissions were collected from 498 to 552 nm. Images were processed with the LAS Image Analysis software (Leica Microsystems).

### Western blots

*B. bronchiseptica* strains were grown for 48 h on BGA plates, transferred to 5 mL SSM liquid culture, and grown overnight. Samples were taken, and the OD_650_ of each sample was matched. After 3-min centrifugation at 13,500 rpm, supernatants for detection of Bsp22 were filtered through a low-binding protein 0.22 µm filter and transferred to new tubes and were concentrated with Amicon Ultra centrifugal filter (molecular weight cutoff 10 kDa). Bacterial pellets were resuspended in PBS to get an OD of 10 to detect flagellin.

The quantity of protein in the supernatants was measured with Qubit Protein Kit. B All samples were boiled for 10 min, sonicated for 5 min, and centrifuged before loading. Proteins were separated by SDS-PAGE 8% and transferred to a PVDF membrane. Bsp22 and flagellin were detected with anti-Bsp22 polyclonal or anti-FlaA serum, respectively, and diluted 1:2,000 in 5% (wt/vol) milk in TBS overnight at 4°C was used (57), followed by anti-mouse IgG conjugated to horseradish peroxidase (1:3,000; Invitrogen) in TBS containing 5% nonfat milk powder at room temperature for 2.5 h. Clarity Western ECL Substrate (Bio Rad #1705060) was used for development according to the manufacturer’s instructions. Samples from each strain were prepared and analyzed independently three times.

### Cytotoxicity assays

J774A.1 macrophages were grown in Dulbecco’s Modified Eagle’s Medium (DMEM) supplemented with 10% (vol/vol) fetal bovine serum (FBS) and 1% penicillin-streptomycin, in a 5% CO_2_ atmosphere at 37°C. Ninety-six-well plates with 1 × 10^5^ cells/well were washed twice with PBS, and 100 µL of bacterial suspensions of *Bb*WT or *Bb*∆*bvgR* on DMEM 10% (vol/vol) FBS was added to the wells in triplicate, at an MOI of 1, 10, and 100. The plates were centrifuged at 300 × *g* for 5 min and then incubated for 4 h in 5% CO_2_ at 37°C. Cytotoxicity was determined by measuring LDH released into a culture medium using Pierce LDH Cytotoxicity Assay Kit (Thermo Scientific), following the manufacturer’s protocols. Bacterial adhesion to J774A.1 cells was determined after 1 h of co-incubation, cells were lysed with 1% v/v Triton X-100, and bacteria were plated in BGA plates. *P*-values were calculated by an unpaired two- or one-tail Student’s *t*-test.

### Murine infection model

Five-week-old outbred CD1 mice were intranasally infected with *B. bronchiseptica*. Bacteria grown overnight on SSM at 36°C were diluted in PBS to obtain an infective dose of 1 × 10^6^ CFU/20 µL. Mice were anesthetized by intraperitoneal injection of ketamine (7.7 mg/kg) and xylazine (0.77 mg/kg) in 0.9% saline, and 10 µL of the bacterial suspension was pipetted directly in each nostril. On days 1, 4, 7, and 21 post-infection, mice were euthanized by intraperitoneal injection of pentobarbital.

Lungs, trachea, and nasal-associated lymphoid tissue were removed aseptically and homogenized for the determination of bacterial burden. Lung weights were recorded before homogenization. Lung homogenates were pelleted, and the supernatants were collected and stored at −80°C. Nasal wash samples were collected by flushing 1 mL of sterile PBS through mice nares. Serial dilutions in PBS of lung homogenate, trachea, and nasal wash were plated on BGA containing streptomycin 200 µg/mL for determination of viable bacteria.

Blood was obtained by cardiac puncture and collected into BD Microtainer Tubes with K_2_EDTA (BD). Complete blood counts were obtained using Hemavet 950FS (Drew Scientific).

### Flow cytometry

Cells isolated from blood, lung homogenates, and nasal washes were analyzed by flow cytometry.

Blood collected in EDTA-containing tubes was treated with RBC lysis buffer (BD Pharm Lyse) with a 15 min-incubation at room temperature to lyse red blood cells. The samples were centrifuged at 1,000 × *g* for 5 min to pellet the cells. Lung homogenate samples were diluted in 4 mL of PBS, filtered through a 100 µm cell strainer and centrifuged at 1,000 × *g* for 5 min. The cells were resuspended in RBC lysis buffer, incubated at 37°C for 2 min, and pelleted again. Samples obtained from the nasal washes were centrifuged at 1,000 × *g* for 5 min.

The pellets containing single cells were resuspended in Fc-block (5 µg in PBS) and incubated for 15 min at 4°C. The samples were pelleted, resuspended in PBS 1% (vol/vol) FBS (PBS-FBS), and mixed with the antibody cocktail. The antibodies used were: CD3e AmCyan, B220 APC-Cy7, CD11b FITC, and Gr-1 PE. After an incubation of 1 h at 4°C in the dark, the cell suspensions were washed with PBS-FBS and resuspended in fixation buffer (0.4% paraformaldehyde) overnight at 4°C. The fixed cells were washed with PBS-FBS and finally resuspended in PBS.

Samples were processed using the LSR Fortessa flow cytometer (BD Biosciences) and analyzed using FlowJo (FlowJo, LLC). Flow cytometry gating strategy for myeloid cells from murine lungs was adapted from Zaynagetdinov et al. (58). Statistical analyses were performed using GraphPad Prism 6, and comparisons between groups were performed by one-way analysis of variance (ANOVA) followed by a Tukey’s multiple comparison test.

### Cytokine quantification

The concentration of cytokines in the lungs homogenates was determined by a quantitative survey analysis using the Meso Scale Discovery V-PLEX Proinflammatory Panel 1 Mouse Kit (IFN-γ, IL-1β, IL-2, IL-4, IL-5, IL-6, IL-10, IL-12p70, KC/GRO, and TNF-α) and the Mouse IL-17 Ultra-Sensitive Kit, per the manufacturer’s instructions. Statistical analyses were performed using GraphPad Prism 6, and comparisons between groups were performed by ANOVA followed by a Tukey’s multiple comparison test.

### Software

Statistical analysis was performed using GraphPad Prism version 9 (GraphPad). Aminoacid sequence alignments were performed using the software MultiAlign. Prediction of solubility was done with CamSol. Biofilm analysis was performed by COMSTAT 2.1 software (59) and ImageJ 1.48h3.

## Data Availability

The mapped reads are available at the Sequence Read Archive (SRA) under BioProject accession number PRJNA1122855.
